# Ecotoxicological Assessment and Biodegradation Potential of Epoxidised Methyl Oleate (EMO) as a Bio-Based Plasticiser in Aquatic Environment

**DOI:** 10.21315/tlsr2025.36.3.12

**Published:** 2025-10-31

**Authors:** Siti Afida Ishak, Asma Liyana Shaari, Noorazah Zolkarnain, Razmah Ghazali, Zulina Abd Maurad

**Affiliations:** Malaysian Palm Oil Board, 6, Persiaran Institusi, Bandar Baru Bangi, 43000 Kajang, Selangor, Malaysia

**Keywords:** Epoxidised Methyl Oleate, Plasticiser, Ecotoxicology, Aquatic Organisms, Biodegradation, Metil Oleat Epoksida, Pemplastik, Ekotoksikologi, Organisma Akuatik, Biodegradasi

## Abstract

Bio-based plasticisers have been developed as sustainable alternatives to phthalate-based plasticisers. However, limited information on their potential ecotoxicological effects on aquatic organisms could hinder their widespread adoption in the market. This study addresses this gap by providing ecotoxicological data on epoxidised methyl oleate (EMO), a potential bio-based plasticiser. This study evaluated the acute toxicity of EMO on five aquatic species (*Moina macrocopa, Daphnia magna, Chlamydomonas reinhardtii, Chlorella vulgaris* and *Macrobrachium lanchesteri*) to develop a species sensitivity distribution (SSD) curve for determining the predicted no-effect concentration (PNEC) of EMO for ecological risk assessment. Additionally, the biodegradation potential of EMO in aquatic environments was assessed using the OECD 301F Manometric Respiratory Test. These results indicate that EMO exhibits a concentration-dependent toxic effect on all tested species. The SSD curve, developed using a normal distribution and a Maximum Likelihood Estimation fit model, yielded 0.359 mg/L for hazardous concentration for 5% of species (HC05). This HC05 value suggests that EMO poses a minimal ecological risk, as it exceeded the water solubility limit (0.012 mg/L). Furthermore, EMO demonstrated favourable biodegradation potential under aerobic conditions. At a concentration of 30 mg/L, EMO achieved 60% biodegradation within four days of incubation, whereas at 100 mg/L, the same level of biodegradation was achieved by Day 11. These findings underscore the importance of assessing the environmental impact of bio-based plasticisers and highlight the EMO’s potential as an eco-friendly alternative to less biodegradable, petroleum-based plasticisers.

## Abstract (fr)

**Abstrak:**

Pemplastik berasaskan bio sedang dibangunkan sebagai alternatif lestari kepada pemplastik berasaskan ftalat. Walau bagaimanapun, kekurangan maklumat mengenai potensi kesan ekotoksikologinya terhadap organisma akuatik boleh menghalang penerimaannya secara meluas di pasaran. Kajian ini dijalankan bagi menangani jurang tersebut dengan menyediakan data ekotoksikologi mengenai metil oleat epoksida (EMO), iaitu pemplastik berasaskan bio. Kajian ini menilai ketoksikan akut EMO terhadap lima spesies akuatik (Moina macrocopa, Daphnia magna, Chlamydomonas reinhardtii, Chlorella vulgaris dan Macrobrachium lanchesteri) untuk membangunkan lengkung taburan kepekaan spesies (SSD) bagi menentukan kepekatan yang tidak memberi kesan yang diramal (PNEC) EMO untuk penilaian risiko ekologi. Selain itu, potensi biodegradasi EMO dalam persekitaran akuatik turut dinilai dengan menggunakan Ujian Respirasi Manometrik OECD 301F. Hasil kajian menunjukkan bahawa EMO mempunyai kesan ketoksikan yang bergantung kepada kepekatan terhadap semua spesies yang diuji. Lengkung SSD, yang dibangunkan menggunakan taburan normal dan model kesesuaian anggaran kemungkinan maksimum, menghasilkan 0.359 mg/L bagi nilai kepekatan berbahaya untuk 5% spesies (HC05). Nilai HC05 ini mencadangkan bahawa EMO mempunyai risiko ekologi yang minimum kerana nilai ini melebihi had keterlarutan air (0.012 mg/L). Tambahan pula, EMO turut menunjukkan potensi biodegradasi yang baik dalam keadaan aerobik. Pada kepekatan 30 mg/L, EMO mencapai 60% biodegradasi dalam masa empat hari inkubasi, manakala pada 100 mg/L, tahap biodegradasi yang sama dicapai pada hari ke-11. Penemuan ini menekankan kepentingan penilaian kesan alam sekitar pemplastik berasaskan bio dan hasil kajian dapat menyerlahkan potensi EMO sebagai alternatif mesra alam kepada pemplastik berasaskan petroleum yang kurang terbiodegradasi.

**HIGHLIGHTS**

- Epoxidised Methyl Oleate (EMO) posed minimal ecological risk with HC05 above its solubility threshold.
- EMO achieved rapid and ready biodegradability under OECD 301F conditions.
- EMO offers a sustainable, low-toxicity alternative to conventional plasticisers.

## INTRODUCTION

The global plasticiser market is expected to grow at a compound annual growth rate (CAGR) of 5.7%, from USD17.0 billion in 2022 to USD22.5 billion in 2027 (Globe Newswire 2023). Plasticisers are essential additives in polymer manufacturing and are primarily used to enhance flexibility, processability and durability by lowering the glass transition temperature and reducing the brittleness of otherwise rigid plastics for diverse applications such as cables, medical devices and packaging (Bocqué *et al*. 2016; Bonifacio *et al*. 2023). Among the types of plasticisers, phthalate-based plasticisers such as di(2-ethylhexyl) phthalate (DEHP), di-n-butyl phthalate (DnBP) and butyl benzyl phthalate (BBP) have dominated the market due to their low cost and high efficiency. However, increasing awareness of the environmental and health risk posed by these substances such as endocrine disruption, reproductive toxicity and developmental effects on both aquatic and terrestrial organisms has led to growing restrictions on their usage (Bodaghi 2020; Bonifacio *et al*. 2023; Lenzi *et al*. 2023). Regulatory frameworks and public health concerns have prompted the development of safer alternatives, particularly in products with direct human contact or environmental exposure (Alhanish & Abu Ghalia 2021).

Bio-based plasticisers are being developed as alternatives to phthalate-based plasticisers and are expected to replace conventional phthalate-based plasticisers. According to Grand View Research (2017), the global demand for bio-based plasticisers was 880 kilotonnes in 2016 and is expected to reach about 1,900 kilotonnes or USD2.68 billion by 2025. Muobom *et al*. (2020) reported a few marketed bio-based plasticisers derived from castor and soybean oil, including GrinstedV® SOFT-N-SAFE and Resiflex® K50. These alternatives are increasingly used in conventional (e.g., PVC) and biodegradable (e.g., PLA) polymers to improve flexibility and reduce reliance on toxic additives. In PLA, the use of natural plasticisers such as vegetable oils, citrates and agro-food waste derivatives has shown promise in enhancing mechanical performance while preserving biodegradability and safety (Righetti *et al*. 2023; Sun *et al*. 2024).

Among the most promising sustainable bioplasticiser are epoxidised of fatty acid esters, particularly those derived from palm oil and other vegetable oils. These compounds including epoxidised fatty acid methyl esters (FAMEs) and isobutyl esters are recognised for their biodegradability, low toxicity, and renewable origins. They can be efficiently synthesised through enzymatic or chemical epoxidation of sustainable feedstocks such as soybean oil or biodiesel (Najera-Losada *et al*. 2025; Sustaita-Rodríguez *et al*. 2021). When incorporated into polymers such as PLA or PVC, these epoxidised esters enhance flexibility, elongation at break, and impact resistance while reducing glass transition temperatures thus overcoming the brittleness of bioplastics (Jadhav *et al*. 2024; Sustaita-Rodríguez *et al*. 2021). Additionally, these bioplasticisers exhibit lower environmental impact and reduced health risks, aligning with increasing regulatory and consumer demand for safer materials (Jadhav *et al*. 2024; Sustaita-Rodríguez *et al*. 2021). The versatility of epoxidised fatty acid esters allows their application across various polymers and industrial uses, including packaging, flooring, and artificial leather. Overall, the development and application of epoxidised fatty acid esters offer a promising direction for sustainable plasticiser technology (Jadhav *et al*. 2024; Kusumaningtyas *et al*. 2022; Najera-Losada *et al*. 2025; Sustaita-Rodríguez *et al*. 2021). Among these epoxidised esters, one promising bioplasticiser is Palm-Flexi Chem, developed from epoxidised palm-based methyl oleate (EMO) by Maurad *et al*. (2019). EMO offers several advantages, including its origin from renewable palm oil resources and its potential to reduce chemical migration in food and beverage packaging. Despite these promising features, comprehensive understanding of EMO’s environmental fate and ecotoxicological impact especially in aquatic ecosystems remains limited. The lack of such data could restrict the regulatory approval and broader commercial adoption of EMO and similar bio-based plasticisers.

To address this gap, this study evaluated the ecotoxicity and biodegradation potential of EMO using a structured ecological risk assessment (ERA) approach. Acute toxicity tests were conducted on representative freshwater species across multiple trophic levels, including primary producers (*Chlamydomonas reinhardtii, Chlorella vulgaris*), primary consumers (*Moina macrocopa, Daphnia magna*), and a secondary consumer (Macrobrachium lanchesteri). A species sensitivity distribution (SSD) curve was constructed to derive the predicted no-effect concentration (PNEC) for EMO, facilitating environmental risk characterisation. Additionally, the biodegradability of EMO was assessed under aerobic conditions following the OECD 301F Manometric Respirometry Test. This study provides important insight into the environmental fate and potential ecological impact of EMO, contributing to its assessment as a safer alternative to conventional phthalate plasticisers. The findings underscore the need to integrate environmental safety evaluations in the development and deployment of bio-based plasticisers to support sustainable and responsible material innovation.

## MATERIALS AND METHODS

### Materials

#### Test organisms

Five aquatic species (*M. macrocopa, D. magna, C. reinhardtii, C. vulgaris* and *M. lanchesteri*) were used as test organisms. These species represent multiple trophic levels, from primary producers to consumers, and were evaluated for endpoints such as acute toxicity and growth inhibition.

*M. macrocopa* and *D. magna* were kept in a 1,000 mL culture medium under controlled conditions, including 12 hours of light and 12 hours of dark photoperiod with a constant temperature of 22 ± 2ºC. The culture medium consisted of aerated tap water with a pH in a range of 7–8 and dissolved oxygen (DO) levels maintained at or above 80%. To ensure the optimal conditions, the culture medium was renewed weekly and M. macrocopa and *D. magna* were fed with a daily diet of *C. vulgaris*.

*C. vulgaris* and *C. reinhardtii* were cultured and maintained in the Conway mineral medium. The cultures were re-cultured weekly in fresh sterilised medium composed of both micro- and macronutrients. This procedure ensured a steady supply of logarithmic and exponential phase cells 5–7 days after inoculation. The pH of the algae medium was adjusted to 7.5 (± 0.01) with 0.1 N NaOH or 0.1 N HCl prior to sterilisation. Algae were incubated in 200 mL conical flasks covered with silicon caps in a 24-h light (10,000–20,000 lx) in incubator (EYELA FLI-2000, Japan) at 22 ± 2°C and shaken at 100 rpm.

*M. lanchesteri* was obtained from a local fish supplier in Selangor, Malaysia and acclimatised for 10 days. The test organism remained in good health throughout this period, with no observed anomalies. The size of the test organisms was maintained within a range of 1.0 ± 0.2 cm. The water temperature was maintained between 22°C and 24°C. The system was brightly lit with indoor lighting for 16 h daily. The test organisms received food daily except for the last 24 h before the test. The DO level in the water was maintained above 80% of the air saturation value.

#### Test substance

**FIGURE 1:**
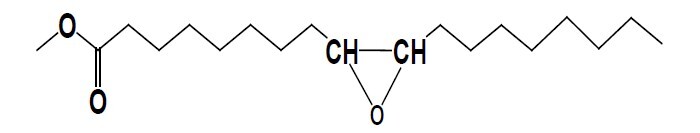
Molecular structure of epoxidised methyl oleate (EMO).

An EMO plasticiser served as the test substance for this study (Fig. 1). The EMO plasticiser is made by epoxidising methyl oleate with acid and peroxide. The produced EMO has an oxirane oxygen content (OOC) value of more than 4.6%, an iodine value (IV) of 2–6 g I_2_/100 g, and a hydroxyl value (OHV) ranging from 14 to 18 mg KOH/g (Maurad *et al*. 2021). The physicochemical properties of EMO, such as water solubility and partition coefficient, were determined using the estimation WSKOW program from Estimation Programs Interface (EPI) Suites^TM^ software developed by the US Environmental Protection Agency (USEPA).

To study the acute toxicity of EMO, which is poorly soluble in water, it was dissolved in methanol, and the final concentration used in all tests was 0.01 mg/L, as recommended by the OECD (Hutchinson *et al*. 006).

### Methods

#### Toxicity assessment

##### M. macrocopa acute toxicity tests

The third generation (F2) of *M. macrocopa* (newly hatched within 24 h) was exposed to different EMO concentrations (0, 0.2, 0.4, 0.8, 1.0 mg/L) for 48 h in triplicates. The immobility of M. macrocopa refers to its inability to resume swimming within 15 sec of gentle agitation. For the acute toxicity test, mortalities were recorded at 24 h and 48 h. Based on the obtained data, the effective concentration that resulted in a 50% mortality rate (EC_50_) was determined.

##### D. magna acute toxicity tests

The F2 generation of *D. magna* (newly hatched within 24 h) was exposed to different EMO concentrations of EMO (0, 0.3, 0.6, 1.2, 1.5 and 2.0 mg/L) for 48 h in triplicates. Ten *D. magna* were used for each replicate. The immobility of D. magna refers to its inability to resume swimming within 15 sec after gentle agitation. For the acute toxicity test, mortalities were recorded at 24 h and 48 h. Based on the obtained data, the effective concentration that resulted in EC_50_ was determined.

Sprague developed a method in 1971 that uses acute toxicity data from bioassays to calculate the safe concentration (SC) of a chemical or substance, such as a plasticiser. It is used to calculate the SC level for a substance that is expected not to cause adverse effects on organisms (Sprague 1971). The SC value was calculated using the following equation:


$$SC=\frac{24h.EC_{50}\times 0.3}{\frac{24h.EC_{50}}{48h.EC_{50}}}$$

Where EC_50_ = effective concentration that resulted in a 50% mortality.

##### Algae growth inhibition test

The effect of EMO on the growth of the green microalgae *C. vulgaris* and *C. reinhardtii* was investigated. Both microalgae were cultured and maintained in Conway mineral medium. The cultures were re-cultured weekly in fresh sterilised medium composed of both micro- and macronutrients. This procedure ensured a steady supply of logarithmic and exponential phase cells 5–7 days after inoculation. The pH of the algae medium was adjusted to 7.5 (± 0.01) with 0.1 N NaOH or 0.1 N HCl prior to sterilisation. Algae were incubated in 200 mL conical flasks covered with silicon caps in a 14 h light (4,000 lx) and 10 h dark cycle incubator (EYELA FLI-2000, Japan) at 22 ± 2°C and shaken at 100 rpm.

The algae growth inhibition test was conducted to assess the effect of plasticiser on microalgae and to determine the 72 h EC_50_ value of EMO plasticiser according to the standard method OECD 201. The exponentially growing cultures of green microalgae *C. vulgaris* and *C. reinhardtii* cultured in Conway media were exposed to five various concentrations of EMO, i.e., 0, 10, 30, 50 and 100 mg/L. The inhibition of growth in relation to a control culture was determined for 72 h. The cell concentration was estimated using a particle counter (Beckman Coulter Z2, USA) (Asma Liyana *et al*. 2024).

##### Prawn acute toxicity test

M. lanchesteri was exposed to different concentrations of the EMO, i.e., 0, 60, 125, 250, 500 and 1,000 mg/L for a 96-hour exposure period. Each concentration was tested in triplicate. Five prawns were placed in a 1,000 mL glass beaker for each replicate. The prawns were placed in a stationary exposure system with the water temperature consistently maintained within the range of 22°C to 24°C. The dissolved oxygen concentration in the water was at least 60% of the air saturation value. Throughout the experiment, mortalities were recorded at 24, 48, 72 and 96 hours. Based on the obtained data, the concentration that resulted in 50% prawn mortality (LC_50_) was determined.

### Statistical Analysis

The statistical analyses were performed using SPSS 27 software. Statistical data are presented as the mean ± standard error. LC_50_/EC_50_ (lethal/effective concentration when 50% of the population has died/affected) and its associated confidence intervals were determined using probit analysis. Probit analysis is a widely used technique for examining dose-response data, enabling the calculation of the concentration at which a specific response occurs and, with the use of 95% confidence intervals, quantifying the uncertainty surrounding this calculation.

#### Species Sensitivity Distribution (SSD) analysis

The SSD analysis was conducted using the SSD Toolbox to evaluate the sensitivity of five aquatic species (*M. macrocopa, D. magna, C. reinhardtii, C. vulgaris* and *M. lanchesteri*) to a tested plasticiser. Toxicity data (e.g., EC_50_ or LC_50_ values) were compiled for all concentrations and expressed in mg/L. A normal distribution was selected to describe the data, and Maximum Likelihood Estimation (MLE) was applied to fit the model. The cumulative distribution of sensitivity was plotted, and the hazard concentration for 5% of the species (HC05) was calculated along with a 95% confidence interval using bootstrapping techniques. The model fit was evaluated using goodness-of-fit tests (iteration 5000), using the SSD Toolbox Software. The SSD curve, HC05, and confidence intervals were used to assess the ecological risks and determine the safe concentration thresholds for the chemical.

#### Biodegradation potential

The OECD 301F Manometric Respiratory Test (OECD 1992) is a commonly used technique to evaluate the biodegradability of chemical substances in an aerobic aquatic environment. It follows OECD guidelines and commonly applied to various organic compounds.

In this study, 30 mg/L of activated sludge from the national sewerage company, Indah Water Konsortium Sdn. Bhd. (IWK) in Kuala Lumpur, Malaysia, was added into a series of biochemical oxygen demand (BOD) bottles along with 30 mg/L EMO, 100 mg/L EMO and 100 mg/L aniline as a positive control. A mineral medium prepared according to OECD guidelines with a total volume of 300 mL in each BOD bottle, was used for the test. A BOD meter (Coulometer, Ohkura Electric Co. Ltd., Japan) was used to directly measure the oxygen uptake in all bottles for 28 days, generating a BOD curve for each test bottle.

The percentage of biodegradation of the test substance was calculated using the following formula:


$$\%biodegradation=\left[\frac{BOD\left(\frac{{mgO}_{2}}{mg\;test\;substance}\right)}{TOD\left(\frac{{mgO}_{2}}{mg\;test\;substance}\right)}\right]\times 100$$

Where:


$$BOD=\left[\left(\frac{{mgO}_{2}}{\begin{array}{l} Litre\;uptake\;by\;test\;and\\ or\;reference\;substance \end{array}}\right)-\left(\frac{{mgO}_{2}}{\begin{array}{l} Litre\;uptake\;by\\ inoculum\;blank \end{array}}\right)\right]\text{mg}/\text{L}$$

Biodegradation is quantified as a percentage of the theoretical oxygen demand (TOD), which is the maximum amount of oxygen required for a complete breakdown of the material under test. The calculation of the TOD value is commonly derived from the molecular formula of a substance. The TOD of the EMO plasticiser was determined using the formula described in Table 1. Oceanographic parameter data processed through satellite imagery.

## RESULTS

### Physicochemical Properties

The physicochemical properties of the plasticiser, particularly the solubility in water and the partition coefficient of water and oil, were studied to determine the fate of this plasticiser in the environment. The physicochemical properties of the EMO plasticiser prediction were based on its chemical structure. The estimated water solubility value of EMO (C_19_H_36_O_3_) is 0.012 mg/L, while the water and oil partition coefficient (Log P_ow_) is 6.175.

### Toxicity Assessment

#### M. macrocopa acute toxicity tests

**FIGURE 2:**
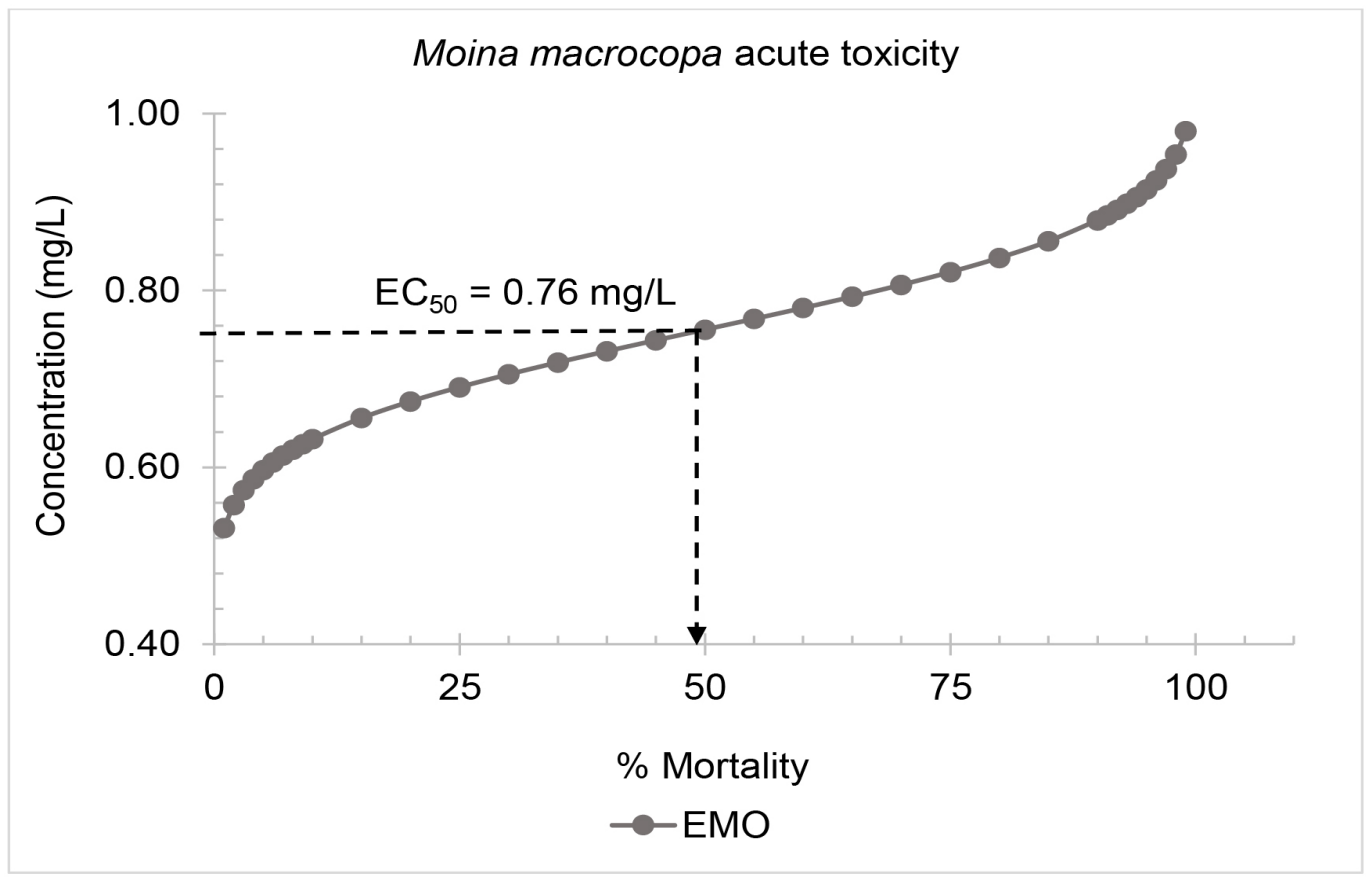
Probit analysis of the epoxidised methyl oleate (EMO) acute toxicity test on *M. macrocopa*.

Acute toxicity 48 h EC_50_ values for the EMO towards M. macrocopa was recorded at 0.76 mg/L (Fig. 2). This value indicates the concentration required to cause 50% mortality in the M. macrocopa population after 48 h of exposure. A control group treated with 0.01 mg/L methanol showed no mortality within the 48 h exposure period, indicating that the solvent methanol itself did not cause any harmful effects on M. macrocopa.

##### D. magna acute toxicity tests

**FIGURE 3:**
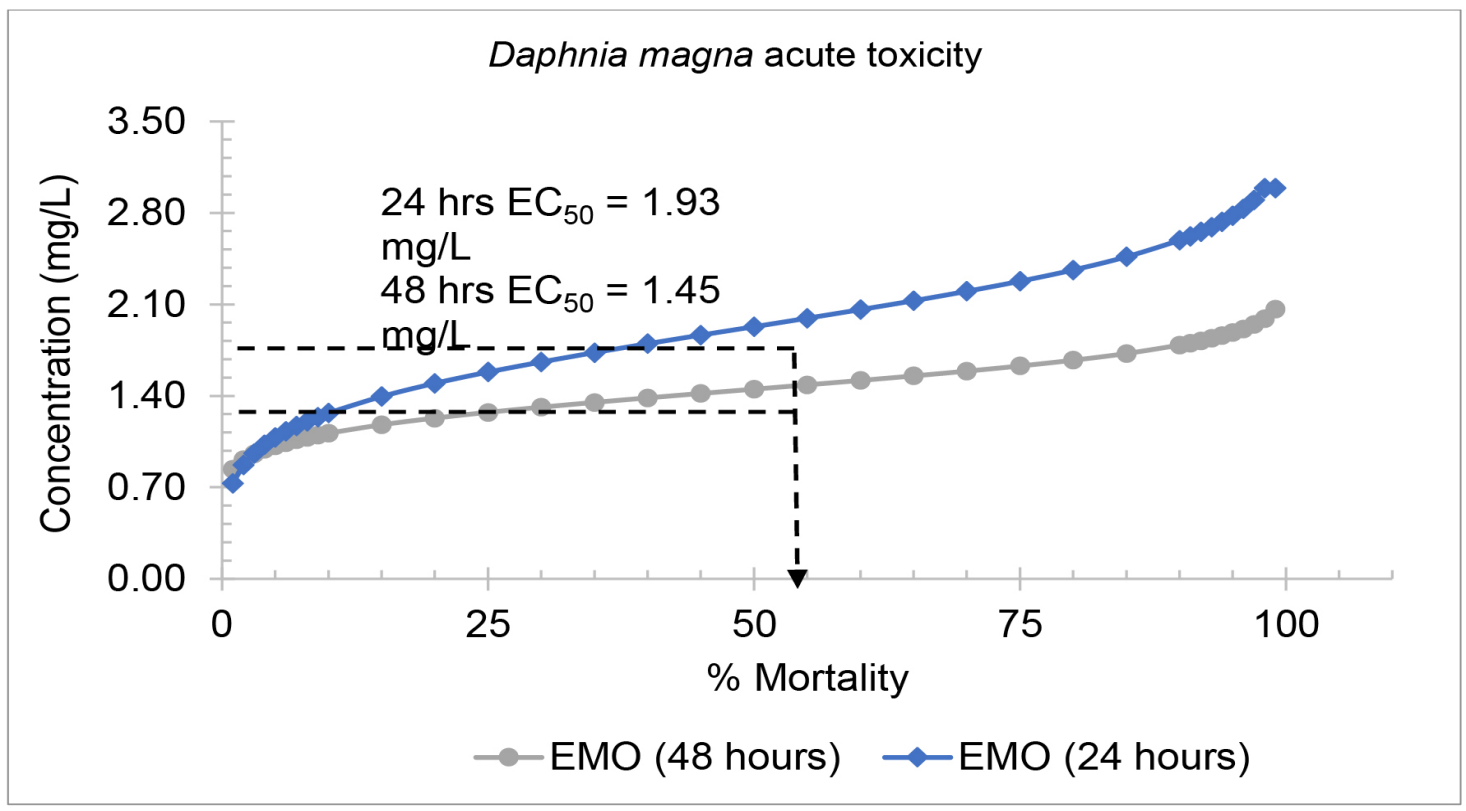
Probit analysis of the epoxidised methyl oleate (EMO) acute toxicity test on *D. magna*.

The results of 24 h and 48 h of *D. magna* acute toxicity test are summarised in Fig. 3. The EC_50_ value after 24 h and 48 h of exposure was calculated to quantify the toxicity of EMO towards *D. magna*. The EC_50_ values, representing the concentration required to cause 50% mortality in the *D. magna* population after 24 h and 48 h of exposure, were 1.93 mg/L and 1.45 mg/L, respectively. The safe concentration of EMO calculated based on the equation is 0.77 mg/L (Table 2).

#### Algae growth inhibition test

**FIGURE 4:**
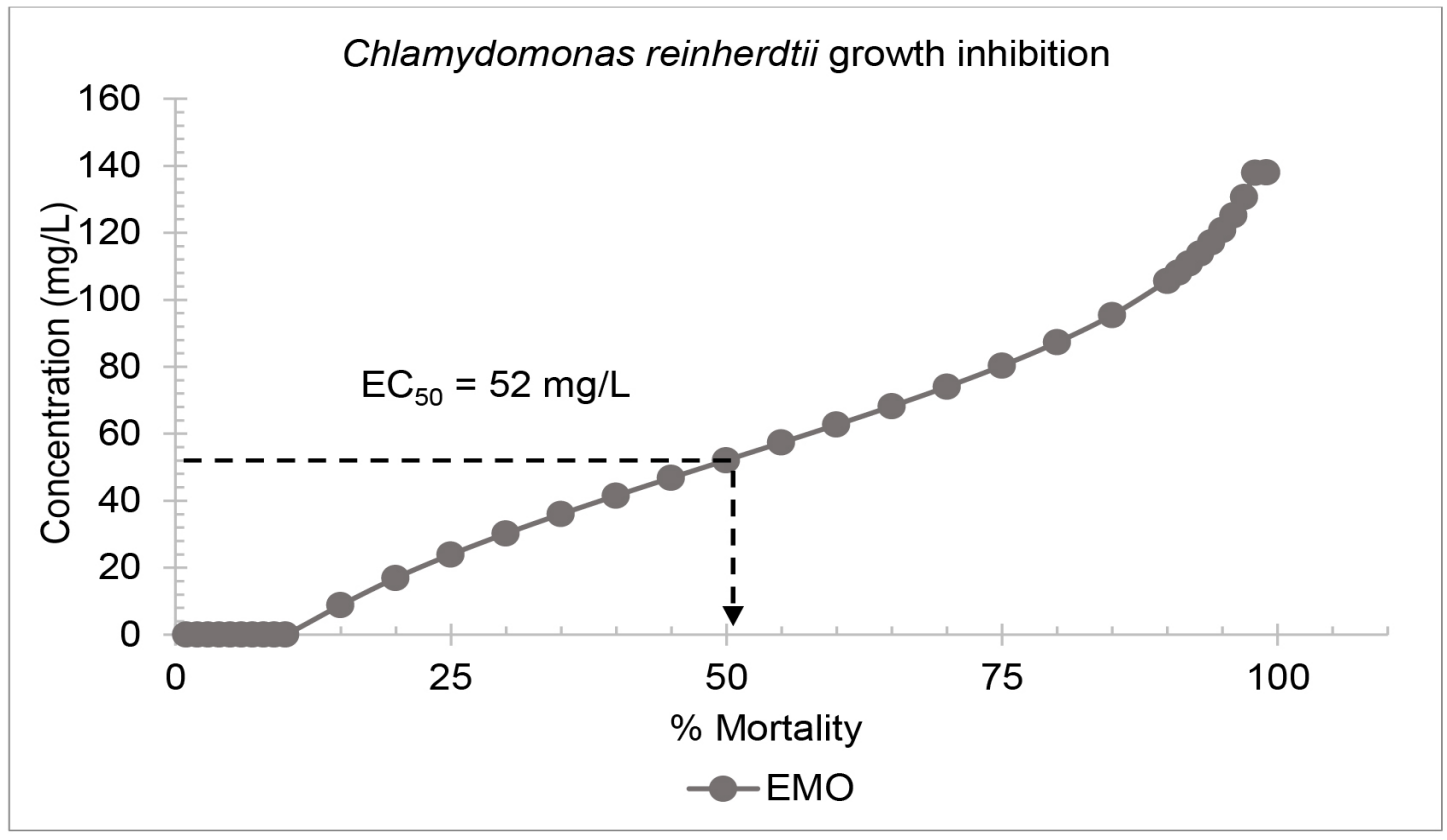
Probit analysis of the epoxidised methyl oleate (EMO) acute toxicity test on *C. reinhardtii*.

**FIGURE 5:**
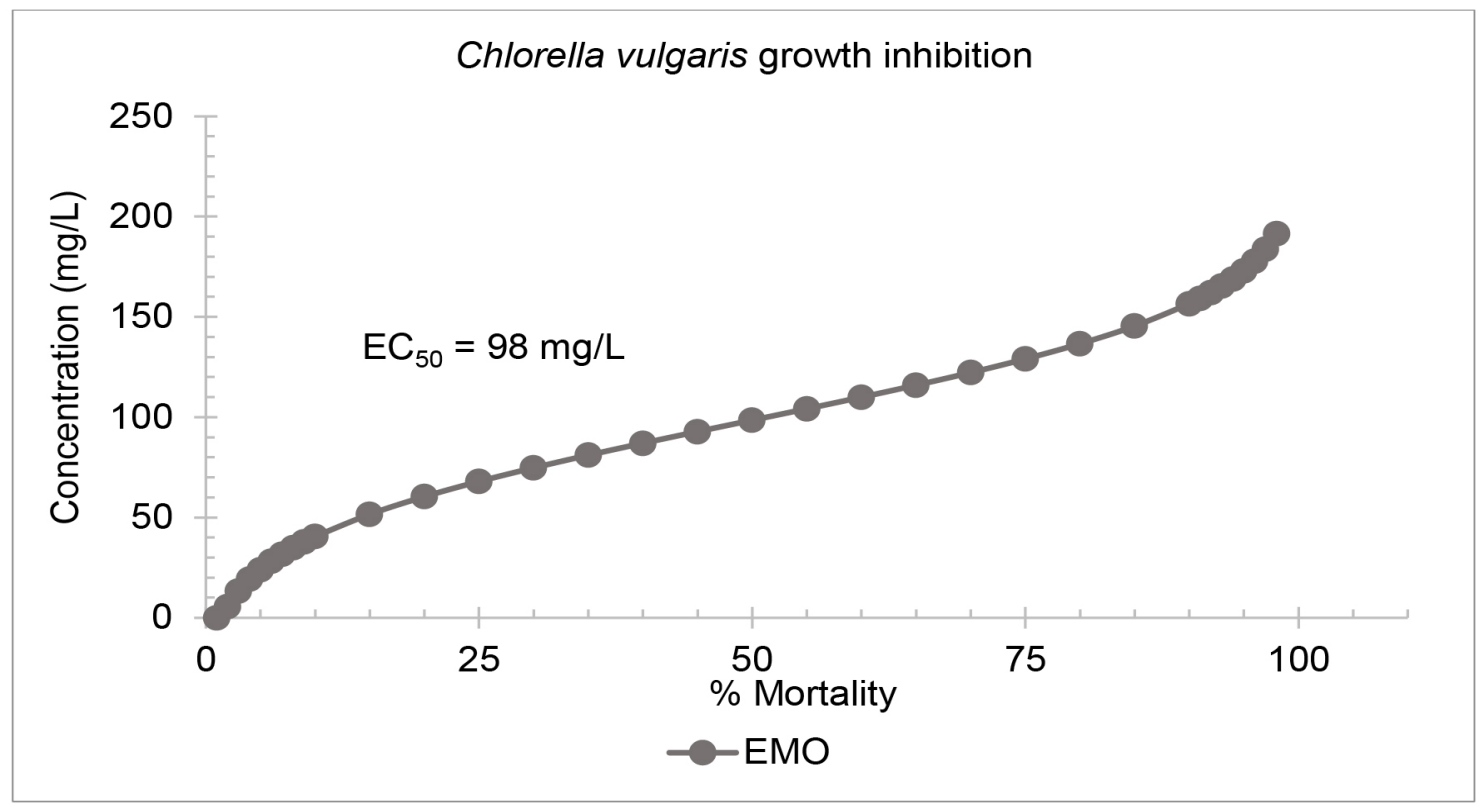
Probit analysis of the epoxidised methyl oleate (EMO) acute toxicity test on *C. vulgaris*.

Growth inhibition tests were conducted on active cultures of *C. reinhardtii* and *C. vulgaris*. The 72-hour EC_50_ of EMO on C. reinhardtii was 52 mg/L (Fig. 4) while for *C. vulgaris*, it was 98 mg/L (Fig. 5). The growth inhibition percentages of both microalgae species increased with increasing plasticiser concentration.

#### Macrobrachium lanchesteri acute toxicity tests

**FIGURE 6:**
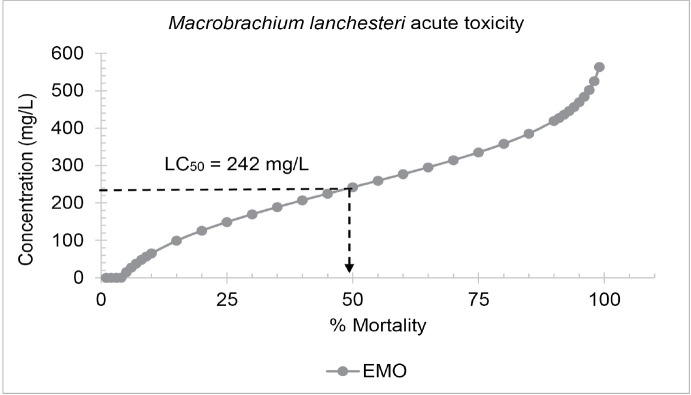
Probit analysis of the epoxidised methyl oleate (EMO) acute toxicity test on *M. lanchesteri*.

The 96-hour LC_50_ values for EMO plasticiser towards higher trophic-level organisms, *M. lanchesteri*, exceeded 100 mg/L (Fig. 6). From this value, it can be suggested that this plasticiser does not affect the mortality of this species. The precipitation of EMO was also observed between 60 mg/L and 1,000 mg/L, as EMO had reached its maximum solubility and could no longer mix with the water due to the solvent carrier used in this test was the highest dosage permitted by the OECD for aquatic toxicity testing.

#### Species Sensitivity Distribution (SSD) analysis

**FIGURE 7:**
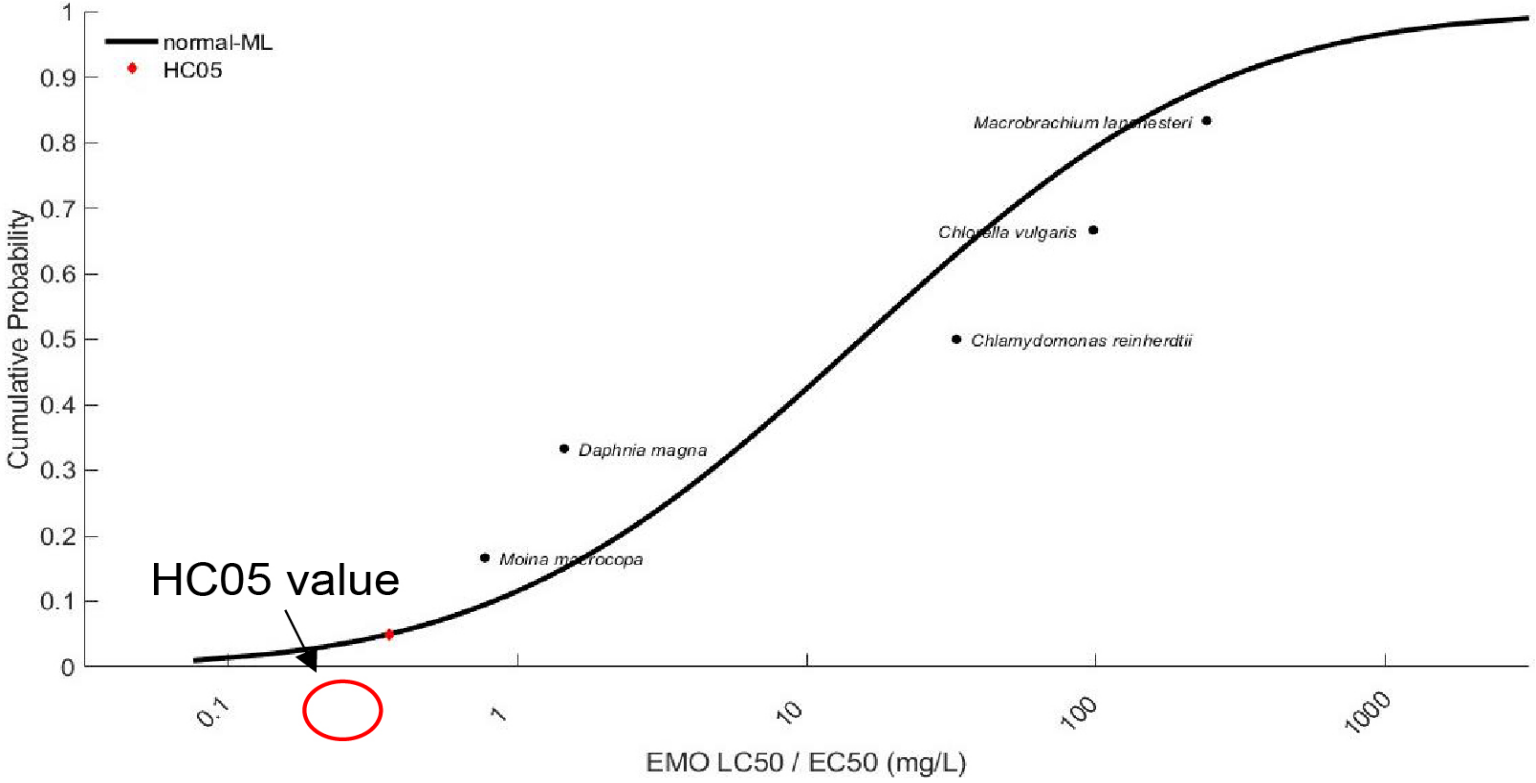
Species sensitivity distributions (SSDs) curve and HC05 values for EMO in freshwater aquatic organisms.

The SSD curve for EMO demonstrated varying sensitivities among aquatic species, with *D. magna* and *M. macrocopa* being the most sensitive, while *M. lanchesteri* showed the highest tolerance (Fig. 7). The HC05 value for EMO was determined to be 0.359 mg/L.

#### Biodegradation potential

The TOD and BOD values (mg/L) after 7, 14, 21 and 28 days of testing are summarised in Table 3. The activated sludge used in the biodegradation test is suitable for use as an inoculum because aniline, a positive control substance, achieved 60% biodegradability on Day 10. A test is considered valid, and the inoculum is considered suitable if the degradation percentage of the aniline reaches the pass level (60%) by Day 14. The BOD values within the incubation medium exhibited a consistent and progressive increase throughout the biodegradation period.

**FIGURE 8:**
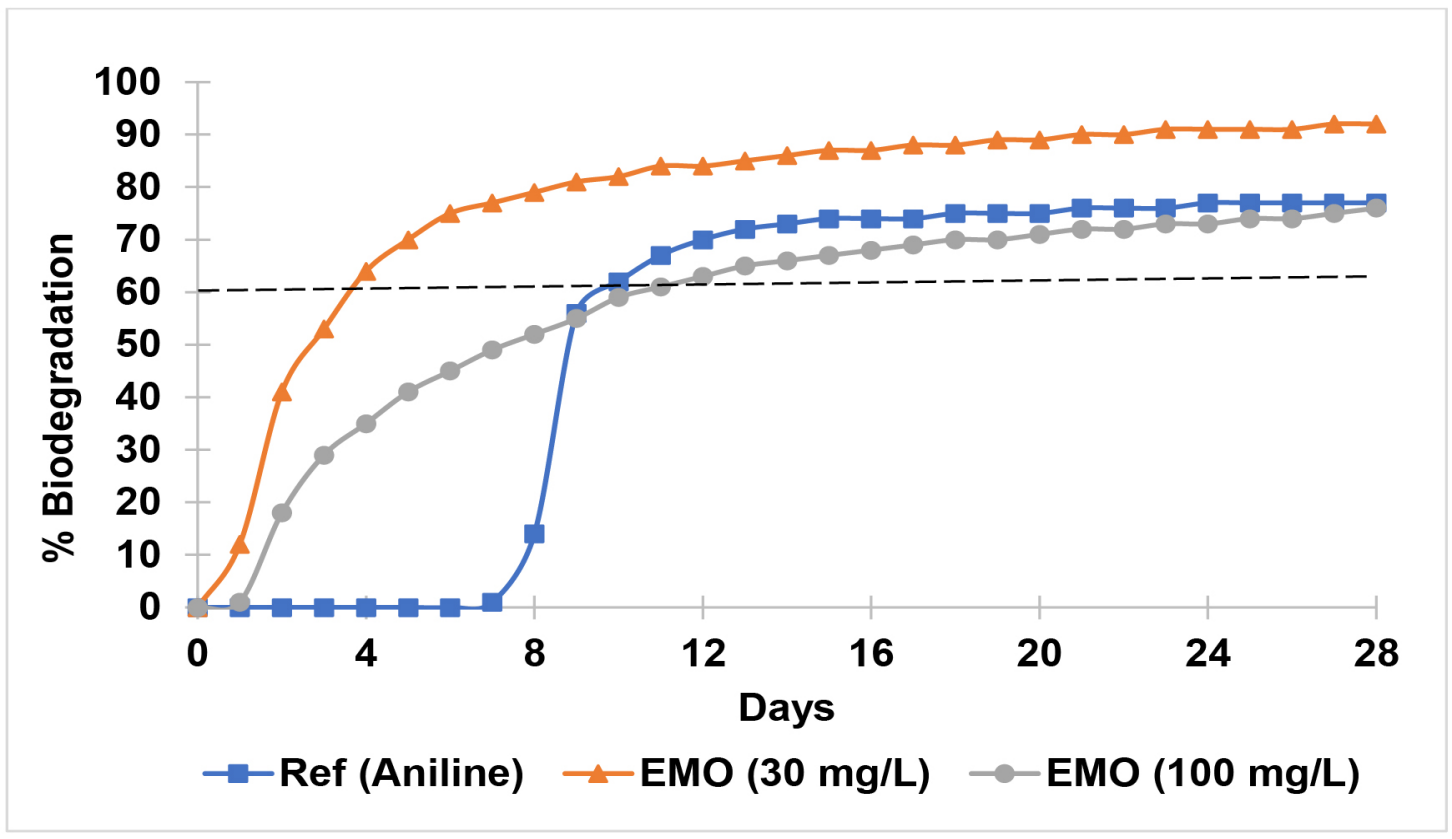
Biodegradation curve of epoxidised methyl oleate (EMO) in aquatic environment.

Over the 28 days test period, EMO exhibited significant biodegradation potential. The curve followed the typical pattern, which indicates that the biodegradation kinetics initially undergone a lag phase, before experiencing an exponential increase, and eventually reached a plateau (Fig. 8). The biodegradation curve of EMO suggests that the microbial community in the activated sludge required only one day to adapt before it could efficiently utilise EMO as a carbon source. The biodegradation of EMO proceeded at a steady rate once the adaptation phase was completed. EMO was found to be readily biodegradable under the conditions applied in the OECD 301F Manometric Respirometry test method, as it reached the pass level (60% biodegradation) within the incubation period (28 days). EMO at a concentration of 30 mg/L achieved 60% biodegradation on Day 4 of the incubation period, whereas EMO at a concentration of 100 mg/L reached 60% biodegradation on Day 11.

### DISCUSSIONS

This study provides a comprehensive ecotoxicological and biodegradation assessment of EMO, a palm-based plasticiser intended as a safer and sustainable alternative to conventional petroleum-based plasticisers. The implications of this study extend to both regulatory frameworks and environmental protection, particularly within tropical freshwater ecosystems. The physicochemical properties of the EMO plasticiser, based on its chemical structure, showed that this plasticiser has a hydrophobic property in water and an affinity for the organic phases to prevent the occurrence of a leaching process from plastic material into water (Beaman *et al*. 2016).

EMO showed concentration-dependent toxicity across aquatic species, with small planktonic crustaceans (*M. macrocopa* and *D. magna*) being the most sensitive organisms tested. This concentration-dependent toxicity is commonly observed with many chemical substances and highlights the potential risks associated with higher concentrations of plasticisers in aquatic environments (Shen *et al*. 2019). The sensitivity of these small planktonic crustaceans towards emerging pollutants is ecologically significant because both species serve as primary consumers and ecologically important species in freshwater food webs. Disruption to their population dynamics can trigger trophic level effects, including algal blooms due to reduced feeding pressure and altered nutrient cycling (Banaee *et al*. 2024; Pisani *et al*. 2022). Crustaceans’ high sensitivity and central role in food webs also make them effective bioindicators for monitoring aquatic ecosystem health (Navarro-Barranco *et al*. 2020). The 24-hour EC_50_ values for M. macrocopa (0.76 mg/L) and D. magna (1.45 mg/L) suggest that EMO exhibits lower acute toxicity than some phthalate esters, such as DEHP which is EC_50_ ≈ 0.56 mg/L towards D. magna (Wang *et al*. 2018), and this acute toxicity concentrations above its solubility limit (0.012 mg/L). According to Staples *et al*. (1997), surface trapping, turbidity and precipitation can interfere with both acute and long-term invertebrate studies regarding the inability to dissolve plasticisers with low water solubility, such as DEHP. As toxicity testing measures the dissolved phase of an emulsion or suspension, it has also been observed that the addition of a solvent carrier, such as methanol, causes the toxicity value of a compound to exceed its true water solubility. These results highlight the importance of considering both nominal and dissolved concentrations in risk assessments of hydrophobic compounds (Bondarenko & Gan 2009). The use of methanol as a carrier solvent may have enhanced EMO’s apparent toxicity due to increased dispersion and partial solubilisation, an artifact often acknowledged in OECD aquatic toxicity protocols (Hutchinson *et al*. 2006). Despite these limitations, the low solubility of EMO inherently reduces its bioavailability in the environment, particularly in dilute systems like rivers and lakes. However, plasticiser accumulation and partitioning to sediments remain plausible exposure routes for benthic organisms (Manatunga *et al*. 2024; Souaf *et al*. 2023). Future studies should investigate chronic sediment toxicity, as crustaceans may be indirectly exposed through ingestion of contaminated detritus or periphyton.

The algal growth inhibition test is a widely used ecotoxicological assay to evaluate the effects of chemicals or environmental stressors on the growth of microalgae. It is a standardised method commonly employed to assess aquatic toxicity under the regulatory guidelines (OECD Guideline 201). Algal species exhibited a wide range of tolerance to EMO exposure. The 72-hour EC_50_ values for *C. reinhardtii* (52 mg/L) and C. vulgaris (98 mg/L) indicate relatively low sensitivity compared to crustaceans, though these concentrations are orders of magnitude above environmental levels. According to Asma Liyana *et al*. (2024), the *C. reinhardtii* and *C. vulgaris* cells did not change in size or form and did not shrink or break in response to the EMO treatments, as observed under a microscope. Nonetheless, these findings are ecologically relevant. Algae are crucial primary producers in aquatic ecosystems, driving oxygen production, carbon fixation, and nutrient cycling. When algal growth is inhibited, even sub-lethally, these ecosystem functions are significantly reduced, especially in environments where pollutants or stressors, such as plasticisers accumulate (Juergens *et al*. 2015; Jüppner *et al*. 2018).

The growth inhibition test results demonstrated that *C. reinhardtii* is more sensitive to the plasticiser EMO compared to *C. vulgaris*, underscoring species-specific differences in metabolic and physiological responses. This increased sensitivity in *C. reinhardtii* is consistent with previous studies showing its susceptibility to photosystem disruption and oxidative stress under xenobiotic exposure, including phthalates and herbicides (Juneau *et al*. 2002). This difference in sensitivity is also likely due to the robust characteristics of *C. vulgaris*, which enable it to adapt more effectively to environmental stressors. *C. vulgaris* is recognised for its notable tolerance to environmental stressors, which is linked to its robust cell wall structure and strong antioxidant defense systems. These features are important for its survival in polluted or challenging environments and should be considered when designing regulatory tests for primary producers (Geng *et al*. 2022; Weber *et al*. 2022).

The freshwater prawn *M. lanchesteri* exhibited high tolerance to EMO, with LC_50_ values exceeding 100 mg/L which is more than the OECD suggested as the maximum test concentration. This indicates that the *M. lanchesteri* population may not be acutely vulnerable by the EMO plasticiser. *M. lanchesteri* has tolerance to this plasticiser, potentially due to the effective metabolic pathways that detoxify or degrade the plasticisers to mitigate acute toxicity effects (Hiltunen *et al*. 2021; Koivisto 1995). This also suggests that acute mortality risks to higher trophic-level organisms may be minimal under realistic exposure scenarios (Handoh & Kawai 2014; Kim *et al*. 2022). Although the LC_50_ value is higher than 100 mg/L, long-term exposure or accumulation of plasticisers in aquatic ecosystems may still adversely affect fish and other organisms (Zanotelli *et al*. 2010). More research is needed to understand the potential chronic effects of plasticisers on M. lanchesteri and the overall health of freshwater ecosystems. Furthermore, prawn such as M. lanchesteri is increasingly recognised as effective bioindicator for detecting chemical stress in freshwater environments, especially where invertebrate diversity is low. Their sensitivity to pollutants is reflected in measurable biochemical, physiological, and molecular changes, making them valuable for environmental monitoring (Jaikumar *et al*. 2021; Jiang *et al*. 2021).

The SSD is a statistical approach to estimate either the concentration of a chemical is hazardous to no more than 5% of all species, or the proportion of species potentially affected by a given concentration of a chemical (Fox *et al*. 2020). The sigmoidal shape of the SSD curve reflects a clear distinction between the sensitive and tolerant species, highlighting the potential adverse effects of low concentrations on the sensitive organisms. From the SSD curve, small crustaceans like *D. magna* and *M. macrocopa* are most sensitive towards EMO, and their appearance can be found at lower parts of the curve as compared to other tested species such as *M. lanchesteri* which showed better tolerance. This relationship is consistent with the findings by Kumari *et al*. (2024), who found that small aquatic organisms tend to be more affected by phthalate-based plasticisers than larger organisms. This sensitivity is attributed to the metabolic ability of large organisms to convert phthalate compounds into less toxic metabolites. The EMO SSD analysis revealed a HC05 at 0.359 mg/L indicating that EMO poses minimal ecological risk, as it exceeded the EMO water solubility concentration (0.012 mg/L). This value significantly exceeds the predicted environmental concentration (PEC) of plasticisers detected in Malaysian freshwater systems, which range from 0.039 μg/L to 10.96 μg/L (Santhi & Mustafa 2013). This broad safety range supports the conclusion that EMO presents negligible acute ecological risk at environmentally relevant concentrations (Santhi & Mustafa 2013).

The biodegradation study was conducted under controlled laboratory conditions that simulated aquatic habitats, a common approach used to evaluate biodegradation processes while addressing several limitations associated with real environmental settings (Zappaterra *et al*. 2023). The biodegradation process was monitored using a BOD system equipped with sensors designed to measure the BOD required by aerobic bacteria to decompose organic matter in each simulated habitat. EMO demonstrated rapid and extensive biodegradation under aerobic conditions, achieving 60% degradation in 4 days at 30 mg/L and in 11 days at 100 mg/L. This relatively high biodegradation rate indicates that EMO can be effectively broken down by microorganisms under aerobic conditions upon disposal into the aquatic environment. These results satisfy the OECD 301F criterion for “ready biodegradability” and underscore EMO’s low persistence in aquatic environments. The short lag phase observed suggests that EMO is readily metabolised by native microbial consortia, likely due to structural similarity to fatty acid esters (Skariyachan *et al*. 2022). The use of microbial consortia, rather than single strains, enhances degradation efficiency due to the complementary metabolic capabilities of different microbes, allowing for the breakdown of both the plasticiser and its additives (Ji *et al*. 2023; Salinas *et al*. 2023). Although direct studies on epoxidised methyl oleate are limited, research on other plasticiser shows that native microbes can adapt to utilise these substances as a sole carbon and energy source and generate nontoxic end products such as carbon dioxide and water, especially when their chemical structures resemble natural lipids (Sharma *et al*. 2024). This rapid biodegradation is promising for the development of environmentally friendly plasticisers that do not accumulate in ecosystems (Ji *et al*. 2023). This is a critical criterion because persistent compounds are more likely to accumulate in sediments, biota or trophic chains, posing long-term ecological threats. EMO’s rapid breakdown reduces its potential for bioaccumulation and trophic transfer (Sharma 2021). The results of this study highlight the favourable biodegradation potential of EMO, making it a promising, environmentally friendly alternative to conventional petroleum-based compounds such as phthalic acid esters (PAEs). PAEs are less biodegradable in sludge at 33%–41% and 50%–62% in 7 and 28 days, respectively (Marttinen *et al*. 2004; Thomas Johnson *et al*. 1984). The easier breakdown of EMO is associated with the accessibility of its ester and epoxide bonds to microbial enzymes, whereas DEHP’s aromatic ring and branched side chains hinder rapid microbial attack (Li *et al*. 2019; Luo *et al*. 2020). Comparatively, other epoxidised vegetable oils such as epoxidised soybean oil (ESO) and epoxidised sunflower oil (ESFO) have also shown similar biodegradation profiles (Prempeh *et al*. 2014; Wu *et al*. 2000), supporting the broader potential of epoxidised bio-based plasticisers as safe alternatives.

The combined ecotoxicological and biodegradation findings suggest that EMO presents a low acute ecological risk at environmentally relevant concentrations. Its rapid degradation, limited bioavailability and comparatively low toxicity to both primary producers and higher trophic organisms support its classification as an environmentally safer plasticiser. These results align with the criteria outlined in international regulatory frameworks such as Registration, Evaluation, Authorisation and Restriction of Chemicals (REACH).

### CONCLUSIONS

This study evaluated the ecotoxicological and biodegradation potential of EMO as a bio-based plasticiser in aquatic environments. Acute toxicity tests revealed that EMO has concentration-dependent toxic effects on freshwater species, with *D. magna* and *M. macrocopa* recognised as the most sensitive organisms. The SSD analysis gave an HC05 value of 0.359 mg/L, suggesting minimal ecological risk as the value exceeded the EMO’s water solubility (0.012 mg/L). Biodegradation studies demonstrated that EMO is readily biodegradable under aerobic conditions, achieving 60% biodegradation within 4 days at 30 mg/L and within 11 days at 100 mg/L. EMO achieved rapid biodegradation under aerobic conditions, satisfying OECD criteria for ready biodegradability. This supports its classification as an environmentally degradable compound. The integration of ecotoxicity and biodegradation findings suggests that EMO offers a safer, more sustainable alternative for use in polymer applications, aligning with green chemistry and regulatory priorities. However, further research is needed to investigate its long-term effects, including chronic toxicity and bioaccumulation in aquatic organisms, to ensure safe application in an environmentally sensitive context. Overall, EMO demonstrates promise as a bio-based plasticiser with low ecological risk and favourable environmental fate, contributing to the ongoing transition toward safer and more sustainable plastic additive.

## Figures and Tables

**TABLE 1:** The theoretical oxygen demand (TOD) calculation for epoxidised methyl oleate (EMO) and aniline (positive control).

Epoxidised methyl oleate (EMO)
C_19_H_36_O_3_ + 26.5 O_2_ → 19CO_2_ + 18H_2_O
26.5 O_2_ / C_19_H_36_O_3_ = 847.97 / 312.49 = 2.71
TOD EMO (30 mg/L) = 9 × 2.71 = 24.4 mg
TOD EMO (100 mg/L) = 30 × 2.71 = 81.3 mg

Aniline (Positive control)

C_6_H_7_N + 8.75 O_2_ → 6CO_2_ + 3.5 H_2_O + NO_2_
8.75 O_2_ / C_6_H_7_N = 279.99 / 93.13 = 3.01
TOD EMO (100 mg/L) = 30 × 3.01 = 90.3 mg

**TABLE 2:** Acute toxicity responses of *D. magna* exposed to EMO.

Time (h)	Regression equation^*^	Correlation coefficient (R^2^)	EC_50_ (mg/L)	Safe concentration (mg/L)
24	y = 2.303x – 2.0492	0.9292	1.93	0.77
48	y = 4.5x – 4.15	0.9907	1.45	

**TABLE 3:** Theoretical oxygen demand (TOD) of epoxidised methyl oleate (EMO) and aniline determined via molecular formula and BOD (mg/L) values.

Plasticiser	Molecular formula	Concentration (mg/L)	TOD	BOD_7_	BOD_14_	BOD_21_	BOD_28_
EMO	C_19_H_36_O_3_	30	2.71 × 9 mg = 24.4 mg	19.6	22.4	23.7	25.4
		100	2.71 × 30 mg = 81.3 mg	40.6	55.2	59.9	63.7
Aniline	C_6_H_7_N	100	3.01 × 30 mg = 90.3 mg	1.6	67.7	70.1	73.5

## References

[b1-tlsr-36-3-235] Alhanish A, Abu Ghalia M (2021). Developments of biobased plasticizers for compostable polymers in the green packaging applications: A review. Biotechnology Progress.

[b2-tlsr-36-3-235] Asma Liyana S, Siti Afida I, Noorazah Z, Zulina AM, Razmah G (2024). Ecotoxicology perspective of epoxidised palm methyl oleate (EPMO): A potential low toxicity plasticizer.

[b3-tlsr-36-3-235] Banaee M, Zeidi A, Mikušková N, Faggio C (2024). Assessing metal toxicity on crustaceans in aquatic ecosystems: A comprehensive review. Biological Trace Element Research.

[b4-tlsr-36-3-235] Beaman J, Bergeron C, Benson R, Cook A, Gallagher K, Ho K, Hoff D, Laessig S (2016). A summary of literature on the chemical toxicity of plastics pollution to aquatic life and aquatic-dependent wildlife.

[b5-tlsr-36-3-235] Bocqué M, Voirin C, Lapinte V, Caillol S, Robin J (2016). Petro-based and bio-based plasticizers: Chemical structures to plasticizing properties. Journal of Polymer Science Part A.

[b6-tlsr-36-3-235] Bodaghi A (2020). An overview on the recent developments in reactive plasticizers in polymers. Polymers Advanced Technologies.

[b7-tlsr-36-3-235] Bondarenko S, Gan J (2009). Simultaneous measurement of free and total concentrations of hydrophobic compounds. Environmental Science and Technology.

[b8-tlsr-36-3-235] Bonifacio A, Bonetti L, Piantanida E, De Nardo L (2023). Plasticizer design strategies enabling advanced applications of cellulose acetate. European Polymer Journal.

[b9-tlsr-36-3-235] Fox DR, van Dam RA, Fisher R, Batley GE, Tillmanns AR, Thorley J, Schwarz CJ, Spry DJ, McTavish K (2020). Recent developments in species sensitivity distribution modeling. Environmental Toxicology and Chemistry.

[b10-tlsr-36-3-235] Geng W, Xiao X, Zhang L, Ni W, Li N, Li Y (2022). Response and tolerance ability of *Chlorella vulgaris* to cadmium pollution stress. Environmental Technology.

[b11-tlsr-36-3-235] Globe Newswire (2023). Plasticizers Global Market Report 2023: Growing demand for plasticizers in construction industry drives sector.

[b12-tlsr-36-3-235] Grand View Research (2017). Bio plasticizers market size worth $2.68 billion by 2025 CAGR 10.7%.

[b13-tlsr-36-3-235] Handoh IC, Kawai T (2014). Modelling exposure of oceanic higher trophic-level consumers to polychlorinated biphenyls: Pollution ‘hotspots’ in relation to mass mortality events of marine mammals. Marine Pollution Bulletin.

[b14-tlsr-36-3-235] Hiltunen M, Vehniäinen ER, Kukkonen JVK (2021). Interacting effects of simulated eutrophication, temperature increase, and microplastic exposure on Daphnia. Environmental Research.

[b15-tlsr-36-3-235] Hutchinson TH, Shillabeer N, Winter MJ, Pickford DB (2006). Acute and chronic effects of carrier solvents in aquatic organisms: A critical review. Aquatic Toxicology.

[b16-tlsr-36-3-235] Jadhav PD, Girase CD, Kulkarni RD, Patwardhan AV, Unnithan UR (2024). Synthesis and properties of a bio-based plasticizer derived from fatty acid methyl ester of erucic acid. Journal of Polymers and the Environment.

[b17-tlsr-36-3-235] Jaikumar IM, Periyakali SB, Rajendran U, Joen-Rong S, Thanasekaran J, Tsorng-Harn F (2021). Effects of microplastics, polystyrene, and polyethylene on antioxidants, metabolic enzymes, HSP-70, and myostatin expressions in the giant river prawn *Macrobrachium rosenbergii*: Impact on survival and growth. Archives of Environmental Contamination and Toxicology.

[b18-tlsr-36-3-235] Ji M, Giangeri G, Usman M, Liu C, Bosaro M, Sessa F, Canu P, Treu L, Campanaro S (2023). An integrated Metagenomic-Pangenomic strategy revealed native microbes and magnetic biochar cooperation in plasticizer degradation. Chemical Engineering Journal.

[b19-tlsr-36-3-235] Jiang Q, Jiang Z, Ao S, Gao X, Zhu X, Zhang Z, Zhang X (2021). Multi-biomarker assessment in the giant freshwater prawn *Macrobrachium rosenbergii* after deltamethrin exposure. Ecotoxicology and Environmental Safety.

[b20-tlsr-36-3-235] Juergens MT, Deshpande RR, Lucker BF, Park JJ, Wang H, Gargouri M, Holguin FO, Disbrow B, Schaub T, Skepper JN, Kramer DM, Gang DR, Hicks LM, Shachar-Hill Y (2015). The regulation of photosynthetic structure and function during nitrogen deprivation in *Chlamydomonas reinhardtii*. Plant Physiology.

[b21-tlsr-36-3-235] Juneau A, El Berdey R, Popovic P (2002). PAM fluorometry in the determination of the sensitivity of *Chlorella vulgaris, Selenastrum capricornutum*, and *Chlamydomonas reinhardtii* to copper. Archives of Environmental Contamination and Toxicology.

[b22-tlsr-36-3-235] Jüppner J, Mubeen U, Leisse A, Caldana C, Wiszniewski A, Steinhauser D, Giavalisco P (2018). The target of rapamycin kinase affects biomass accumulation and cell cycle progression by altering carbon/nitrogen balance in synchronized *Chlamydomonas reinhardtii* cells. The Plant Journal.

[b23-tlsr-36-3-235] Kim L, Cui R, Kwak IJ, An YJ (2022). Sub-acute exposure to nanoplastics via two-chain trophic transfer: From brine shrimp *Artemia franciscana* to small yellow croaker *Larimichthys polyactis*. Marine Pollution Bulletin.

[b24-tlsr-36-3-235] Koivisto S (1995). Is Daphnia magna an ecologically representative zooplankton species in toxicity tests?. Environmental Pollution.

[b25-tlsr-36-3-235] Kumari A, Rajput S, Raina P, Chaudhary G, Kaur R, Kumari A, Rajput VD, Mandzhieva SS, Minkina T, Hullebusch V (2024). The ubiquity of microplastics and phthalates in aquatic ecosystems and toxicological concerns. Emerging contaminants.

[b26-tlsr-36-3-235] Kusumaningtyas RD, Prasetiawan H, Anggraeni ND, Anisa EDN, Hartanto D (2022). Conversion of free fatty acid in *Calophyllum inophyllum* oil to fatty acid ester as precursor of bio-based epoxy plasticizer via SnCl2-catalyzed esterification. Polymers (Basel).

[b27-tlsr-36-3-235] Lenzi L, Degli Esposti M, Braccini S, Siracusa C, Quartinello F, Guebitz GM, Puppi D, Morselli D, Fabbri P (2023). Further step in the transition from conventional plasticizers to versatile bioplasticizers obtained by the valorization of levulinic acid and glycerol. ACS Sustainable Chemistry and Engineering.

[b28-tlsr-36-3-235] Li J, Zhang J, Yadav MP, Li X (2019). Biodegradability and biodegradation pathway of di-(2-ethylhexyl) phthalate by *Burkholderia pyrrocinia* B1213. Chemosphere.

[b29-tlsr-36-3-235] Luo X, Chu H, Liu M (2020). Synthesis of bio-plasticizer from soybean oil and its application in poly(vinyl chloride) films. Journal of Renewable Materials.

[b30-tlsr-36-3-235] Manatunga DC, Sewwandi M, Perera KI, Jayarathna MD, Peramune DL, Dassanayake RS, Ramanayaka S, Vithanage M (2024). Plasticizers: Distribution and impact in aquatic and terrestrial environments. Environmental Science: Processes and Impacts.

[b31-tlsr-36-3-235] Marttinen SK, Hänninen K, Rintala JA (2004). Removal of DEHP in composting and aeration of sewage sludge. Chemosphere.

[b32-tlsr-36-3-235] Maurad ZA, Bakar ZA, Hazmi ASA, Idris Z (2019). Palm-based plasticiser.

[b33-tlsr-36-3-235] Maurad ZA, Idris Z, Hazmi ASA, Ismail R, Bakar ZA, Chooi FC, Yee LW (2021). Epoxidized palm-based methyl oleate plasticizers, (PI 2016702567/PCT/MY2017/050039) granted MY183650A, Malaysia.

[b34-tlsr-36-3-235] Muobom SS, Umar AMS, Brolin AP, Soongseok Y (2020). A review on plasticizers and eco-friendly bioplasticizers: Biomass sources and market. International Journal of Engineering Research & Technology.

[b35-tlsr-36-3-235] Najera-Losada L, Narváez-Rincón PC, Orjuela A, Gomez-Caturla J, Fenollar O, Balart R (2025). Plasticization of polylactide using biobased epoxidized isobutyl esters derived from waste soybean oil deodorizer distillate. Journal of Polymers and the Environment.

[b36-tlsr-36-3-235] Navarro-Barranco C, Ros M, Tierno de Figueroa JM, Guerra-García JM, Lovrich G, Thiel M (2020). Marine crustaceans as bioindicators: Amphipods as case study. Fisheries and aquaculture.

[b37-tlsr-36-3-235] OECD (1992). OECD guidelines for the testing of chemicals. Section 3: Degradation and accumulation. Test 301: Ready biodegradability.

[b38-tlsr-36-3-235] Pisani XG, Lompré JS, Pires A, Greco LL (2022). Plastics in scene: A review of the effect of plastics in aquatic crustaceans. Environmental Research.

[b39-tlsr-36-3-235] Prempeh N, Li J, Liu D, Das K, Maiti S, Zhang Y (2014). Plasticizing effects of epoxidized sun flower oil on biodegradable polylactide films: A comparative study. Polymer Science Series A.

[b40-tlsr-36-3-235] Righetti GIC, Nasti R, Beretta G, Levi M, Turri S, Suriano R (2023). Unveiling the hidden properties of tomato peels: Cutin ester derivatives as bio-based plasticizers for polylactic acid. Polymers (Basel).

[b41-tlsr-36-3-235] Salinas J, Carpena V, Martínez-Gallardo MR, Segado M, Estrella-González MJ, Toribio AJ, Jurado MM, López-González JA, Suárez-Estrella F, López MJ (2023). Development of plastic-degrading microbial consortia by induced selection in microcosms. Frontiers in Microbiology.

[b42-tlsr-36-3-235] Santhi VA, Mustafa AM (2013). Assessment of organochlorine pesticides and plasticisers in the Selangor River basin and possible pollution sources. Environmental Monitoring and Assessment.

[b43-tlsr-36-3-235] Sharma I, Murillo-Tovar MA, Saldarriaga-Noreña H, Saeid A (2021). Bioremediation techniques for polluted environment: Concept, advantages, limitations, and prospects. Trace metals in the environment: New approaches and recent advances.

[b44-tlsr-36-3-235] Sharma K, Nayarisseri A, Singh SK (2024). Biodegradation of plasticizers by novel strains of bacteria isolated from plastic waste near Juhu Beach, Mumbai, India. Scientific Reports.

[b45-tlsr-36-3-235] Shen C, Wei J, Wang T, Wang Y (2019). Acute toxicity and responses of antioxidant systems to dibutyl phthalate in neonate and adult *Daphnia magna*. PeerJ.

[b46-tlsr-36-3-235] Skariyachan S, Taskeen N, Kishore AP, Krishna BV (2022). Recent advances in plastic degradation: From microbial consortia-based methods to data sciences and computational biology driven approaches. Journal of Hazardous Materials.

[b47-tlsr-36-3-235] Souaf B, Methneni N, Beltifa A, Turco VL, Danioux A, Litrenta F, Sedrati M, Mansour HB, Di Bella G (2023). Occurrence and seasonal variation of plasticizers in sediments and biota from the coast of Mahdia, Tunisia. Environmental Science and Pollution Research.

[b48-tlsr-36-3-235] Sprague JB (1971). Measurement of pollutant toxicity to fish-III. Water Research.

[b49-tlsr-36-3-235] Staples CA, Adams WJ, Parkerton TF, Gorsuch JW, Biddinger GR, Reinert KH (1997). Aquatic toxicity of eighteen phthalate esters. Environmental Toxicology and Chemistry.

[b50-tlsr-36-3-235] Sun S, Weng Y, Zhang C (2024). Recent advancements in bio-based plasticizers for polylactic acid (PLA): A review. Polymer Testing.

[b51-tlsr-36-3-235] Sustaita-Rodríguez A, Vega-Rios A, Bugarin A, Ramos-Sánchez VH, Camacho-Dávila AA, Rocha-Gutiérrez B, Chávez-Flores D (2021). Chemoenzymatic epoxidation of highly unsaturated fatty acid methyl ester and its application as poly(lactic acid) plasticizer. ACS Sustainable Chemistry and Engineering.

[b52-tlsr-36-3-235] Thomas Johnson B, Heitkamp MA, Jones JR (1984). Environmental and chemical factors influencing the biodegradation of phthalic acid esters in freshwater sediments. Environmental Pollution Series B, Chemical and Physical.

[b53-tlsr-36-3-235] Wang Y, Wang T, Ban Y, Shen C, Shen Q, Chai X, Zhao W, Wei J (2018). Di-(2-ethylhexyl) phthalate exposure modulates antioxidant enzyme activity and gene expression in juvenile and adult *Daphnia magna*. Archives of Environmental Contamination and Toxicology.

[b54-tlsr-36-3-235] Weber S, Grande PM, Blank LM, Klose H (2022). Insights into cell wall disintegration of *Chlorella vulgaris*. PLoS ONE.

[b55-tlsr-36-3-235] Wu X, Zhang X, Yang S, Chen H, Wang D (2000). The study of epoxidized rapeseed oil used as a potential biodegradable lubricant. Journal of the American Oil Chemists’ Society.

[b56-tlsr-36-3-235] Zanotelli VRT, Neuhauss SCF, Ehrengruber MU (2010). Long-term exposure to bis(2-ethylhexyl) phthalate (DEHP) inhibits growth of guppy fish *(Poecilia reticulata)*. Journal of Applied Toxicology.

[b57-tlsr-36-3-235] Zappaterra F, Renzi M, Piccardo M, Spennato M, Asaro F, Di Serio M, Vitiello R, Turco R, Todea A, Gardossi L (2023). Understanding marine biodegradation of bio-based oligoesters and plasticizers. Polymers (Basel).

